# Cryo-EM Grid Preparation of Membrane Protein Samples for Single Particle Analysis

**DOI:** 10.3389/fmolb.2018.00074

**Published:** 2018-07-31

**Authors:** Germán G. Sgro, Tiago R. D. Costa

**Affiliations:** ^1^Departamento de Bioquímica, Instituto de Química, Universidade de São Paulo, São Paulo, Brazil; ^2^Department of Life Sciences, Imperial College London, MRC Centre for Molecular Microbiology and Infection, London, United Kingdom

**Keywords:** protein purification, membrane protein complex, vitrification, cryo-EM, single particle analysis

## Abstract

Recent advances in cryo-electron microscopy (cryo-EM) have made it possible to solve structures of biological macromolecules at near atomic resolution. Development of more stable microscopes, improved direct electron detectors and faster software for image processing has enabled structural solution of not only large macromolecular (megadalton range) complexes but also small (~60 kDa) proteins. As a result of the widespread use of the technique, we have also witnessed new developments of techniques for cryo-EM grid preparation of membrane protein samples. This includes new types of solubilization strategies that better stabilize these protein complexes and the development of new grid supports with proven efficacy in reducing the motion of the molecules during electron beam exposure. Here, we discuss the practicalities and recent challenges of membrane protein sample preparation and vitrification, as well as grid support and foil treatment in the context of the structure determination of protein complexes by single particle cryo-EM.

## Introduction

During the course of the past 10 years, spectacular advances have been made in the ability to solve macromolecular structures using cryo-EM, culminating in the 2017 Nobel Prize in Chemistry awarded to Jacques Dubochet, Joachim Frank and Richard Henderson for developing the technique and applying it to high-resolution structure determination of biomolecules in solution (Cheng et al., 2017). One significant development that made the recent breakthroughs possible was the introduction of direct electron detectors with superior DQE (Detective Quantum Efficiency) (Milazzo et al., 2011; Bammes et al., 2012), which can also operate at higher frame rates allowing recording of movies instead of single images. These features permit correction of the specimen movement caused by the electron radiation and temperature-changes induced drifts during the subsequent image processing steps (frame alignment) (Glaeser et al., 2011; Brilot et al., 2012; Li et al., 2013; Zheng et al., 2017).

In spite of these advances, it is still difficult to routinely obtain high-resolution structures of single proteins or their complexes. Many aspects of protein sample preparation are still poorly understood, and therefore difficult to master. Here, we present an overview of the recent developments in protein preparation methods for cryo-EM, to facilitate the understanding of protein behavior and assist the user during this process.

## Protein sample preparation and stabilization

**Figure 1:**
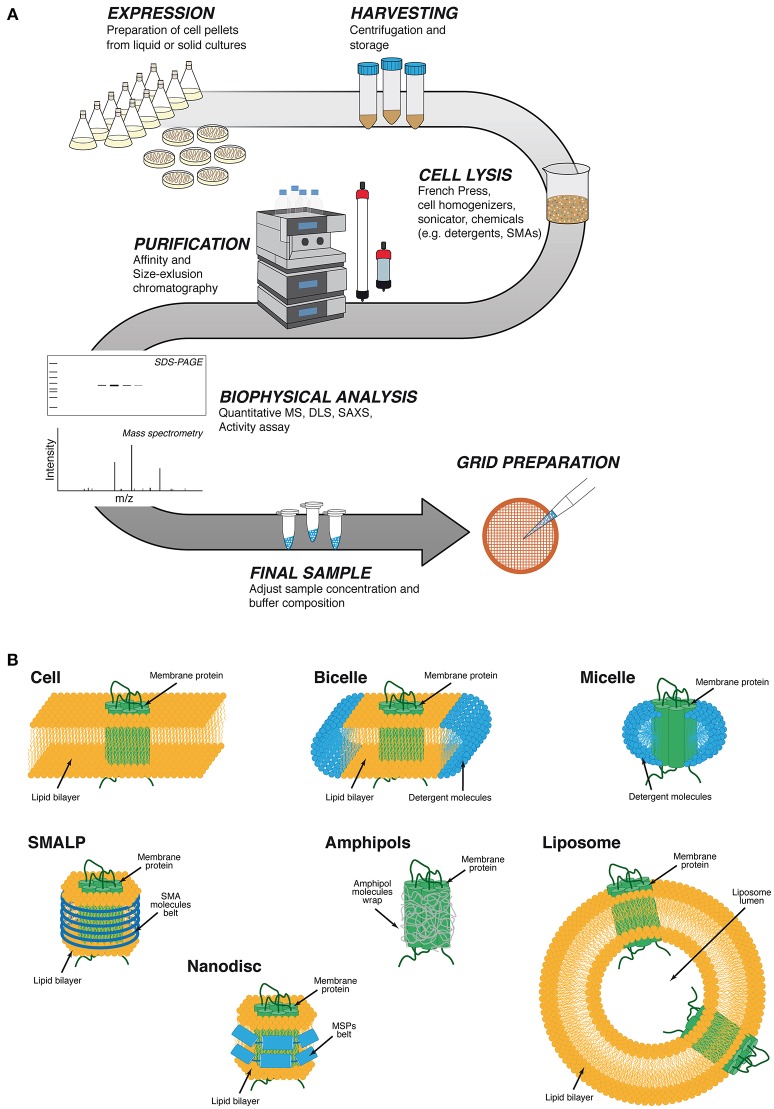
Generic protein purification workflow and different membrane protein stabilization strategies using artificial membranes. **(A)** Cytoplasmic or membrane proteins are initially expressed in liquid or solid cultures, and pellets are stored after harvesting by centrifugation. Different physical or chemical cell disruption methods are utilized for releasing cytoplasmic proteins into solution or to obtain cell membrane extracts. Impure cytoplasmic proteins or solubilized cell membranes containing the protein of interest are purified by combination of different fast protein liquid chromatography (FPLC) methods. After protein stability, integrity and activity is verified by various biophysical techniques. The final sample concentration and buffer composition are adjusted before EM grid preparation. **(B)** Protein transmembrane domains are protected by the hydrophobic cell membrane phospholipid acyl chains. Micelles are spherical vesicles in which the detergent hydrophobic chains face inward and the hydrophilic polar heads face outward. Bicelles are obtained by a mixture of lipids and short chain detergents. The lipids will interact with the protein to form a lipid bilayer and the detergent will form the rim of the bicelle. Micelles will form after the solubilization of the membrane protein by detergents. SMALP (styrene-maleic acid lipid particles) are polymeric nanoparticles that protect the acyl chain of the lipid bilayer. Nanodiscs are lipid bilayers stabilized by wrapping a belt of amphipathic helix-rich membrane scaffold proteins (MSPs) around the detergent-solubilized membrane proteins. Amphipol polymers wrap around the hydrophobic patches of the membrane protein to form a stable complex in solution. Liposomes are artificial spherical lipid membranes where membrane proteins can assemble.

Before freezing the specimen on the EM grid, it is important to evaluate several biochemical and biophysical aspects of the protein sample, such as composition, purity, homogeneity, stability, and biochemical activity (Figure 1A). Prior knowledge of the protein molecular weight and oligomeric state(s), and buffer composition (salt concentration, pH, co-factors, cryo-protectants and other additives) in which the protein is stable can remarkably facilitate cryo-EM grid preparation (Figure 1A). Additionally, evaluation of sample homogeneity by negative staining electron microscopy (NS-EM) before cryo-EM grid preparation will help not only to validate that the correct purification protocol has been followed, but will also ensure that no contaminants or degradation products are present in the protein sample. Such contaminants can interfere significantly with the subsequent computational analyses of the particle images.

## Membrane protein sample preparation

It is estimated that 20–30% of the genes in almost all known (eubacterial, archaeal and eukaryotic) genomes encode membrane proteins (Wallin and Heijne, 1998; Krogh et al., 2001). Membrane proteins play important roles in cells and organelles, affecting the function of tissues or the behavior of organisms (Alberts et al., 2014). Structural information is indispensable for understanding the biological mechanisms in which these proteins play critical roles. Yet, the determination of three-dimensional structures of membrane proteins represents the most challenging cases of all proteins, mainly due to seemingly insurmountable difficulties during sample preparation.

The main challenge in membrane protein or membrane protein complex biochemistry is, by far, the determination and optimization of the chemical conditions capable of solubilizing the protein from the membrane and stabilizing its native state in solution. Unfortunately, a method that works for one particular protein may not be suitable for another; therefore there is no “golden rule” to efficiently stabilize any membrane protein, and an empirical trial-and-error process is currently the best way to proceed. In any case, understanding the physicochemical properties and pros and cons associated with the different methods can be a useful starting point for deciding which strategy may work best for a particular case.

For many years, detergents have conventionally been used to solubilize membrane proteins or protein complexes, enabling their stable handling in solution (Seddon et al., 2004; Privé, 2007; Paulsen et al., 2015). Detergents solubilize membrane proteins by mimicking the natural lipid bilayer environment of membranes, and can be classified according to their structure into four major categories; Ionic, Non-ionic, Zwitterionic detergents and bile acid salts; (Seddon et al., 2004; Figure 1B). While using detergents, some aspects have to be considered during the process. Proteins must be handled in solutions containing detergent above the critical micelle concentration (CMC) in order to minimize denaturation. It should be kept in mind that protein solubilization does not always maintain their native structure and stability; thus, a detergent that is used for extraction from the membranes may not be compatible with subsequent stabilization steps and/or biochemical characterization of the solubilized protein.

Recently, a new class of solubilizing agents has been designed, consisting of a mixed copolymer with a hydrophilic backbone and hydrophobic side chains, known as amphipols (Popot et al., 2011). These molecules wrap around the hydrophobic portion of the protein and expose their hydrophilic components to the aqueous environment (Figure 1B). Amphipols have significant advantages over traditional detergents. For example, as they are completely associated with the protein, there is little or no free polymer in solution; thus minimizing problems related to phase separation encountered in crystallization, increased viscosity in nuclear magnetic resonance spectroscopy (NMR) experiments, or diminished contrast in cryo-EM images. Amphipols have successfully been used in structural studies of many membrane proteins demonstrating their efficacy as solubilizing agents (Flötenmeyer et al., 2007; Althoff et al., 2011; Bai et al., 2015; Mazhab-Jafari et al., 2016; Wilkes et al., 2017).

Although membrane proteins solubilized using these agents show significant stability and solubility, they are still confined to an environment very different from the natural lipidic membrane. It has been broadly shown that membrane composition is critical for the correct functioning of membrane-associated proteins, by modulating their structure and stability via specific lipid-protein interactions (Zhou and Cross, 2013; Saliba et al., 2015). A solution to overcome these drawbacks is reconstructing the protein into artificial lipid membranes, like liposomes (Rigaud and Lévy, 2003; Figure 1B). Liposomes have provided good results not only in the determination of membrane protein structures by cryo-EM (Tilley et al., 2005; Wang and Sigworth, 2009; Jensen et al., 2016; Kudryashev et al., 2016), but are also a useful tool for analyzing these proteins by NMR (Warschawski et al., 2011; Dürr et al., 2012).

Because of the intrinsic difficulties of purifying and manipulating liposomes, in recent years the most commonly used lipid bilayer environment-based tool is the nanodisc (Denisov and Sligar, 2016, 2017). Nanodiscs are composed of a region of the membrane lipid bilayer wrapped by amphipathic helix-rich membrane scaffold proteins (MSPs) (Bayburt et al., 2002), resulting in a disc-shaped stable particle that contains the target protein or protein complex (Figure 1B). The strong interactions between MSPs and membrane lipids and the very low solubility of the latter in water permits the self-assembly of nanodiscs (Denisov and Sligar, 2017). Although this technology has successfully been used in membrane protein structure determination by different methodologies, cryo-EM may be the one where the advantages of nanodiscs usage is most effectively utilized (Efremov et al., 2015; Gatsogiannis et al., 2016; Kedrov et al., 2016), sometimes improving the resolution and quality of the structures obtained in other studies (Gao et al., 2016; Shen et al., 2016).

Another detergent-free solubilizing tool is styrene–maleic acid copolymers (SMAs) (Dörr et al., 2016). The most striking feature of these amphipathic molecules is their ability to solubilize lipid bilayers directly from cells as polymer-surrounded (instead of MSP-surrounded) nanodiscs (Long et al., 2013; Figure 1B). To date, this technique has been used in several biochemical and biophysical reports (Orwick et al., 2012; Dörr et al., 2014; Lee et al., 2016), but in only a few structural studies of membrane proteins (Postis et al., 2015; Parmar et al., 2018).

## Cryo-EM grid preparation

**Figure 2:**
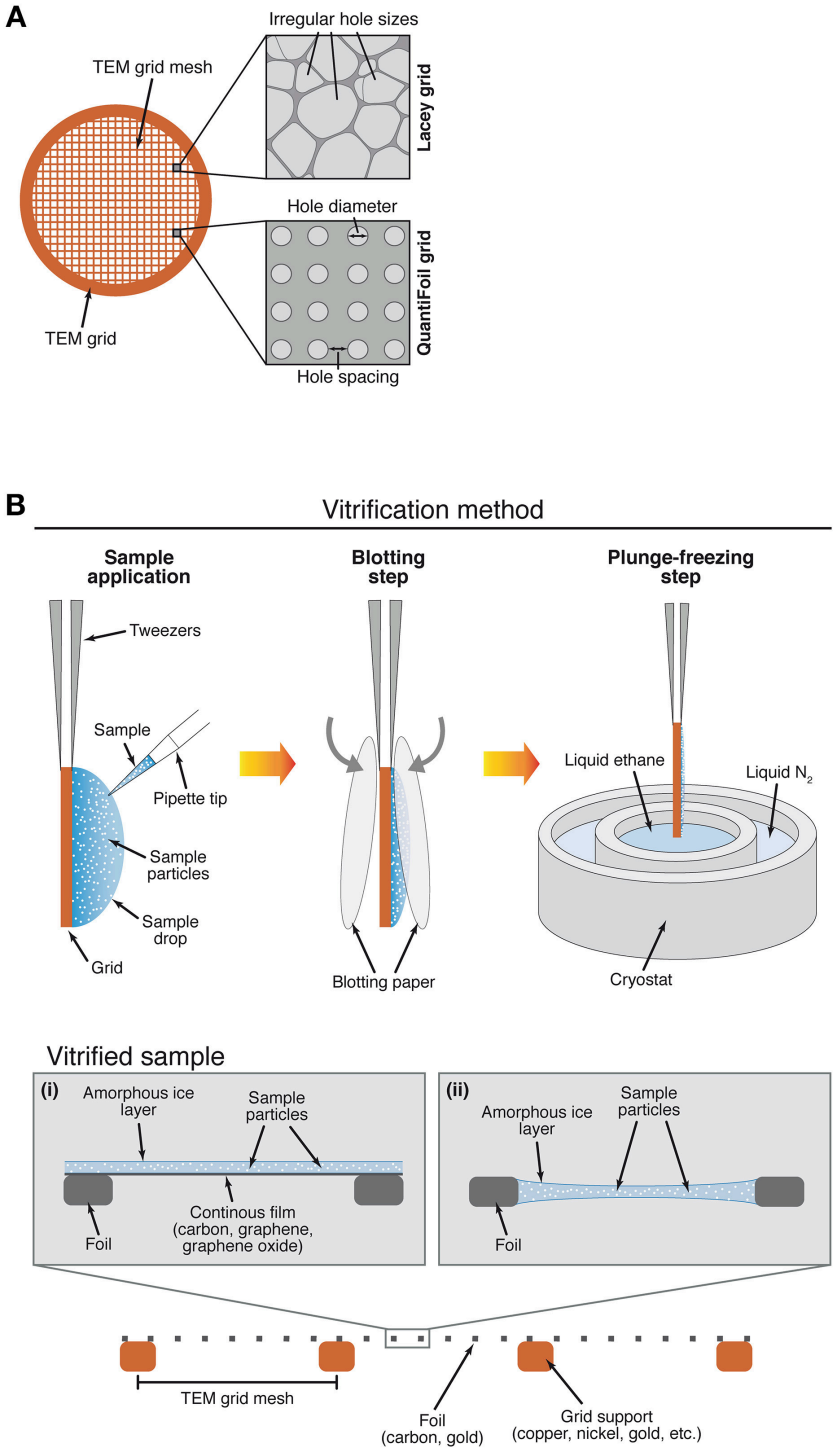
Different designs of a TEM (transmission electron microscopy) grid and semi-automated method for specimen vitrification. **(A)** Examples of a TEM grid with irregular hole size foil (Lacey) or with defined hole diameter and spacing (Quantifoil). **(B)** An automated plunge-freezing device is commonly used for specimen vitrification. Sample is applied with a pipette at the surface of the cryo-EM grid and sample excess is removed by blotting with filter paper, followed by immediate freezing in liquid ethane. The specimen can be frozen on a grid with (i) or without (ii) a thin continuous film made of different materials. TEM grids with different grid mesh, foil and grid support materials can be used during specimen freezing.

Cryogenic sample-grid preparation allows fixing biological samples by rapidly transferring and cooling them in liquid ethane (−188°C). Under these conditions, ice crystals are unable to form, thus preserving the specimen integrity. Ideally, cryo-EM samples should be contained in a thin layer of vitreous ice, with a thickness as close as possible to the dimensions of the particles. This minimizes multiple scattering events and maximizes sample contrast in the microscope. In practice, approximately 3 μL of sample is deposited on the cryo-EM grid (Figure 2A) to promote its absorption followed by blotting with filter paper to remove the excess of liquid and create a very thin layer of protein suspension on the grid, which is then rapidly frozen (plunge freezing) in liquid ethane (Figure 2B). Unfortunately, optimal ice thickness is difficult to reproduce from one grid to another due to the uneven surface properties of the blotting paper. In order to solve this limitation, alternative blotting-free methods that ensure a more reliable and reproducible grids preparation have recently been developed. The same newly developed automated systems have also reduced the protein sample volume required for each grid preparation from the microliter to the nano- or even femtoliter range. Some of the new devices include the “Spotiton” robot that uses an inkjet dispenser to deposit 2–16 nL droplets onto self-blotting grids (Jain et al., 2012; Razinkov et al., 2016; Noble et al., 2018), a spray-plunging system that delivers droplets directly onto the EM grid (Feng et al., 2017), a microcapillary-based system that applies and spread the sample on the grid (Arnold et al., 2017), and a system that uses surface acoustic waves to deliver 30–200 fL droplets from a microfluidic chip to the EM grid (Ashtiani et al., 2018).

It is often the case that when applied to an EM grid (Figure 2A) the protein complex aggregates and/or falls apart. This may occur because the specimen is exposed to physical conditions different to the ones inside the cell or the optimized purification buffer (Figure 2B). Surfaces such as amorphous carbon, metal support structure, filter paper and air-water interface affect the way particles behave and are distributed on the grid (Figure 2B). Notably, if we consider a 3-mm diameter grid and an 800-Å-thick ice layer, the surface area to volume ratio in this layer is 4 orders of magnitude greater than in the original 3-μL drop of solution containing the sample, adding even more complexity to the process. Efforts are being made to understand and surpass these detrimental effects on biological samples (Glaeser and Han, 2017). Together, all of these factors can lead to a failure of grid preparation, or an overall lack of reproducibility from grid to grid.

Often, the protein concentration on the grid does not match the protein concentration in solution due to surface adhesion effects. Particle density can be higher than expected if they tend to absorb more to the surface, and lower if they are repelled from the surface or absorbed to the support structures. Taking into account that we need 5–10 times more sample concentration for cryo-EM than for NS-EM, a very helpful table that provides an estimation of the number of particles expected in the thin layer of vitreous ice on holey grids for a given concentration of sized macromolecular complexes can be found in the 2016 review by Vinothkumar and Henderson (2016).

Several modifications and alternatives have been tested to overcome limitations in particle stability, distribution and (preferential) orientation during cryo-EM grid preparation. In cases of dynamic complexes, glycerol gradient centrifugation coupled to chemical cross-linking (GraFix; Kastner et al., 2008) has been proven to increase sample stability. The addition of detergents below its CMC (Lyumkis et al., 2013; Fernandez-Leiro et al., 2015), the use of affinity grids or antibodies (Kelly et al., 2008; Earl et al., 2017), chemically oxidized carbon films (Llaguno et al., 2014), PEGylation of gold grids (Meyerson et al., 2014) and the use of a “DNA cage” that surrounds and protects proteins (Martin et al., 2016a) are some of the most successful approaches described to improve stability, distribution and orientation of particles on the cryo-EM grid. In cases where only small sample volumes can be obtained, direct blotting from native gels to EM grids has also been proven successful (Knispel et al., 2012). It is worth mentioning specimen tilting as a method of overcoming particle preferential orientation, a concept that is not new and that recently has been explored again with good results (Naydenova and Russo, 2017; Tan et al., 2017).

## Supports and foils

The grid is a 3-mm diameter piece of metal mesh that serves as the major support for the sample. Grids are specified in units of metal lines per inch; thus grids of mesh 200 or 400 contain 200 or 400 lines per inch, respectively (Figure 2A). The foil is the thin layer directly located across and on top of the grid support that contains holes or some other sophisticated geometry in it (Figure 2B). A regular repeating array of circular holes (Quantifoil or C-Flat grids) are usually the first type of grids chosen because they facilitate automated data collection. Alternatively, grids with irregular geometry such as Lacey carbon can also be used (Figure 2A).

Grid supports and foils (Figure 2B) can be made of different materials. For example, metals (alone or in alloys) are used for making the grid supports, i.e., copper, nickel, molybdenum, silicon, titanium, aluminum and gold (Vonck, 2000; Yoshioka et al., 2010; Russo and Passmore, 2014b). Copper is the most common because it is cheap and a good conductor (an important feature to avoid detrimental sample charging). In the case of the foil, by far the most common material is amorphous carbon (Figure 2B) because it is inert, relatively electron transparent, electrically (somewhat) and thermally conductive, and is easily manufactured into foils. Many other materials (TiSi, SiN, SiO_2_, SiC) have also been considered and tested over the years for different reasons, but only a few of them have given good results and only in particular cases (Typke et al., 2004; Rhinow and Kühlbrandt, 2008; Yoshioka et al., 2010). Amorphous carbon foils have their own limitations when used in conjunction with a different grid support material. Two main reasons have encouraged researchers to test different materials both in grid supports and foils in order to overcome the drawbacks of using two-material grids. The first one is derived from differential thermal contraction. When a traditional (copper/carbon or gold/carbon) grid is cooled to liquid N_2_ temperatures (−196°C), the metal support shrinks more than the carbon foil causing the wrinkling of the latter (“cryo-crinkling”) and subsequent loss of tension (Glaeser, 1992; Booy and Pawley, 1993). The coefficient of thermal expansion for copper is 16.6 × 10^−6^ K and for carbon is 0.5–8.8 × 10^−6^ K (Glaeser, 1992; Booy and Pawley, 1993). Cryo-crinkling promotes a movement of 200–400 Å perpendicular to the plane of the support (Russo and Passmore, 2014b) as well as lateral movements in the horizontal plane (Brilot et al., 2012) during electron irradiation. By making the thermal expansion coefficient of the grid support closer to that of the foil (e.g., using titanium, molybdenum or tungsten instead of copper or gold), one can minimize this effect (Booy and Pawley, 1993; Fujiyoshi, 1998; Vonck, 2000). The second challenge arises from the electrical properties of carbon, as its conductivity is quite poor and it behaves as a semi-conductor (Larson et al., 2011). As with other semi-conductors, conductivity decreases (or resistivity increases) as temperature decreases. This promotes build-up of charges on the foil and consequent radiation-induced movement that causes loss of resolution in electron micrographs (Russo and Passmore, 2016a; Russo and Henderson, 2018). In the last few years, gold has become an excellent alternative for overcoming these issues (Russo and Passmore, 2014b, 2016b). First, since the entire grid structure (both support and foil) is made of the same material, it shrinks uniformly and the flatness/rigidity of the foil is maintained after cryo-plunging. Second, resistivity in thin gold films is several orders of magnitude lower than for thin carbon films, and it is more conductive as temperature decreases (normal behavior for metals), helping to reduce accumulation of charges on the supports (Russo and Passmore, 2016b). The improved stability of the support reduces particle movement during image collection by more than an order of magnitude, leading to improved image quality.

## Foil treatments

The supporting surfaces are often hydrophobic, which prevents the efficient spreading of aqueous solution onto grids. In order to reduce hydrophobicity, they are treated with low energy plasmas, which are created by ionization of a low-pressured gas aka “glow discharge”. Air is the most used gas mixture, but there are also plasma chambers that use defined mixtures of gases including oxygen, hydrogen and argon, or amylamine-enriched atmosphere. Air provides the grid with a negative charge, while amylamine yields a positive charge, both being beneficial in a case-dependent manner. The ions also interact with the surface removing certain contaminations. A common problem in cryo-EM protein sample preparation is incomplete wetting of the grid surface, which can be solved by adjusting the plasma and conditions to achieve a more uniform spreading of the solution on the grid (Figure 2B). Other foil treatments tested in the past include UV (Burgess et al., 2004) and electron (Miyazawa et al., 1999) radiation.

Creating conditions that promote the partition of protein particles into the holes of the foil is sometimes a significant challenge. One possible strategy is to add another surface (a continuous film) on top of the foil in order to provide an extra physical support for the particles to adsorb to. In practice, additional films may also help to overcome limitations such as low protein concentration, particle distribution in the grid holes, preferential orientation and air-water interface issues (Figure 2B). As shown in the literature, the most common type of film is a very thin layer of amorphous carbon (10–100 Å), which is relatively simple to make and use in the laboratory (Bernal and Stock, 2004; Passmore and Russo, 2016).

Unfortunately, amorphous carbon films contribute substantially to background signal, which is less of an issue when studying large protein complexes, but becomes significant for 150-kDa (or smaller) complexes. In these cases, an alternative is to use a different type of film. Graphene (Pantelic et al., 2011), for example, is an excellent support material because it is a 1-atom thick (0.34 nm) hexagonal lattice of carbon atoms with extremely good conductivity properties (Heersche et al., 2007; Chen et al., 2008) and mechanical strength (Lee et al., 2008; Wang et al., 2009; Figure 2B). It is also effectively invisible at the resolutions reached in electron microscopy (Meyer et al., 2007). Hence, although graphene supports are difficult to make and transfer (Li et al., 2009; Regan et al., 2010; Pantelic et al., 2011), they have more ideal properties that potentially may reduce the effects of charging and improve image quality. Graphene is naturally hydrophobic and must be rendered hydrophilic to allow the wetting of the surface. A number of methods have been recently developed to overcome this limitation. Partial hydrogenation of the graphene surface has been used to control protein adsorption to the surface (Russo and Passmore, 2014a). Also, graphene oxide (Pantelic et al., 2010) has gained popularity (Bokori-Brown et al., 2016; Boland et al., 2017), because it is more easily produced and deposited on grids (Martin et al., 2016b), and because it is hydrophilic by nature thus obviating the plasma treatment step (Figure 2B). Still, reproducibility and coverage using graphene oxide is difficult and it can contribute to background noise.

## Conclusions

The practicalities, challenges and examples described above, along with numerous other studies, illustrate how intricate and difficult protein sample preparation can be. Determination of high-resolution structures by cryo-EM is a rapidly growing field, in particular with the recent developments in detection and recording, user-friendly microscopes and better preforming software. In this context, protein sample preparation still remains a trial and error process, where different approaches have to be explored in order to maximize the chances for success.

The ultimate goal is to move from trial-and-error processes to more controlled and reproducible protein sample preparation protocols. Better grid supports will reduce specimen movement during data collection, diminish build-up of charge and help control the orientation and distribution of particles within the ice layer.

We anticipate future advances such as the design and production of other automated vitrification devices based on different technologies, the development of rapid ice thickness screening protocols, the minimization of radiation-induced motion and charging, and the exploitation of more tunable interacting surfaces. Additionally, we will witness in the near future an increased investment in the development of new solubilization methods for membrane protein sample preparation, improved electron microscope detectors, recording hardware and data processing software. These new developments will allow us to reach the theoretical resolution limit of this powerful technique sooner than expected.

## Author contributions

GS wrote the manuscript and prepared the figures. TC edited the manuscript and figures, and supervised the work. Both authors approved the final version of the manuscript.

### Conflict of interest statement

The authors declare that the research was conducted in the absence of any commercial or financial relationships that could be construed as a potential conflict of interest.
